# Development and Preclinical Testing of a Novel Neurodenervant in the Rat: C3 Transferase Mitigates Botulinum Toxin’s Adverse Effects on Muscle Mechanics †

**DOI:** 10.3390/toxins17050234

**Published:** 2025-05-09

**Authors:** Cemre Su Kaya Keles, Zeynep D. Akdeniz Dogan, Can A. Yucesoy

**Affiliations:** 1 Institute of Biomedical Engineering, Boğaziçi University, 34684 Istanbul, Turkey; 2 Institute of Structural Mechanics and Dynamics in Aerospace Engineering, University of Stuttgart, 70569 Stuttgart, Germany; 3 Department of Plastic, Reconstructive, and Aesthetic Surgery, Marmara University, 34854 Istanbul, Turkey

**Keywords:** botulinum toxin type A, C3 transferase, spasticity management, muscle force, range of motion, extracellular matrix stiffness

## Abstract

Spasticity, characterized by elevated muscle tone, is commonly managed with botulinum toxin type A (BTX-A). However, BTX-A can paradoxically increase passive muscle forces, narrow muscles’ length range of force exertion (l_range_), and elevate extracellular matrix (ECM) stiffness. C3 transferase, known to inhibit myofibroblast and fascial tissue contractility, may counteract ECM stiffening. This study investigated whether combining BTX-A with C3 transferase reduces active forces without altering passive forces or l_range_. Additionally, we examined the isolated effects of C3 transferase on muscle levels. Male Wistar rats received injections into the tibialis anterior (TA): Control (*n* = 7, saline) and C3 + BTX-A (*n* = 7, 2.5 µg C3 + 0.1U BTX-A). TA forces were measured one month post-injection, and isolated C3 transferase effects were assessed in separate groups (Control and C3, *n* = 6 each). Active forces were 43.5% lower in the C3 + BTX-A group compared to the Control group. No differences between groups in passive forces (*p* = 0.33) or l_range_ (*p* = 0.19) were observed. C3 transferase alone had no significant effect on relative muscle mass (*p* = 0.298) or collagen content (*p* = 0.093). Supplementing BTX-A with C3 transferase eliminates BTX-A’s adverse effects at the muscle level. C3 transferase alone causes no atrophy or collagen increase, which are key factors in BTX-A-induced ECM stiffening. This novel neurodenervant formula shows promise for advancing spasticity management.

## 1. Introduction

Cerebral palsy (CP), the most common childhood-onset disability [1], results from brain damage or malformation during development. A major neurological feature of CP [2] is spasticity, characterized by elevated muscle tone and exaggerated stretch reflexes, which lead to significant and lifelong motor dysfunctions [3]. Botulinum toxin type A (BTX-A) is widely employed in the management of spasticity in CP [3]. By reducing muscle tone and allowing the target muscle to stretch, commonly through BTX-A administration, the goal is to minimize the development of muscle contractures [4]. Along with reduced muscle tone, the expectation is to eventually decrease passive resistance and improve motion in the joint [3,5,6] to facilitate independent movement in one’s daily life. Given that the muscles exposed to BTX-A remain the motor for joint movement, it is crucial to understand the mechanical and structural effects induced by the toxin at the muscular level. Although numerous studies have addressed such effects [7,8,9], fewer have focused on assessing those in direct relation to the main expected benefits of the administration of BTX-A.

Experiments in animals assessing the short-term effects showed that exposure to BTX-A either fails to increase the target muscle’s length range of force exertion (l_range_) [10] or may even narrow it [11,12]. As l_range_ is a metric directly determining the muscle-actuated joint range of motion, BTX-A’s effects are functionally highly important. Moreover, BTX-A was shown to consistently lead to increased muscle passive forces and higher intramuscular collagen content [11,13], which are directly relevant to the passive resistance in the joint. The need for an improved understanding of BTX-A effects becomes more prominent in the long term [4,14]. Finite element analyses were conducted to investigate the underlying mechanisms of BTX-A’s effects on muscular mechanics [15] and how those mechanisms are altered over the treatment course [16]. These studies indicated that via muscle fiber–extracellular matrix (ECM) mechanical interactions, the stiffness of the ECM plays a central role in determining the experimental findings that are counter-indicated compared to the main expected benefits of BTX-A administration. Experimental studies confirmed modeled anticipations, demonstrating a consistent increase in muscle passive force, indicating elevated ECM stiffness in the long term and a sustained or even more pronounced narrowing of l_range_ [17,18]. Note that model findings also highlighted the persistence of such adverse BTX-A effects post-treatment, based on a retained increased ECM stiffness. In light of these previous studies, we suggest that mitigation strategies for the central effect of ECM stiffening are extremely important to benefit from the spasticity management effect of BTX-A while avoiding counter-indicated effects on the mechanics of exposed muscles.

Certain studies showed that C3 transferase (botulinum toxin type C3) has the capacity to inhibit specific signaling pathways, suggesting that it may assist the management of cellular Rho functions [19]. A cell culture study utilizing C3 transferase as a Rho-associated kinase inhibitor showed its inhibitory effect on the contractile activity of human myofibroblasts [20]. Additionally, certain research demonstrated the efficacy of C3 transferase in inhibiting contractile activity in fascial tissues [21]. Our novel proposal is the following: C3 transferase, showing promise in inhibiting myofibroblast and fascial tissue contractility, may be relevant for controlling ECM stiffness changes. If validated, we conceptualized that the use of C3 transferase can help limit ECM stiffness increases and, therefore, diminish the adverse effects of BTX-A on muscular mechanics.

We aimed to investigate, for the first time, the effects of a combined administration of BTX-A and C3 transferase on skeletal muscle mechanics. We specifically tested the hypotheses that combined administration of BTX-A and C3 transferase into the rat tibialis anterior (TA) muscle causes (i) decreased active muscle forces while yielding no change in (ii) passive muscle forces and (iii) the muscle’s l_range_. Additionally, we examined the isolated effects of C3 transferase on muscle mechanics and structure.

## 2. Results

### 2.1. Study 1: Effects of Combined Administration of BTX-A and C3 Transferase on Muscle Mechanics

**Figure 1:**
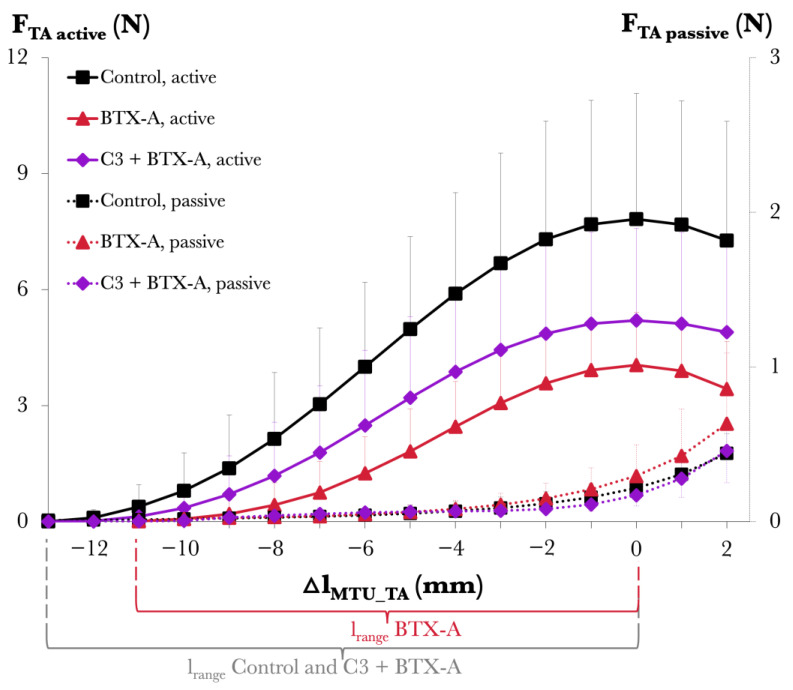
Forces of the tibialis anterior (TA) against its increasing muscle–tendon unit length (l_MTU_) in Study 1. Active and passive muscle forces are shown as mean values with standard deviations for the Control and C3 + BTX-A groups. The l_MTU_ of the TA is expressed as a deviation from its optimum length (Δl_MTU_TA_ = 0 mm). Data concerning the BTX-A group were extracted from our earlier publication [17] to facilitate comparison and interpretation of the findings.

Figure 1 shows the TA muscle’s length–force characteristics.

Passive muscle forces. The ANOVA results, with muscle–tendon unit lengths (l_MTU_) of the TA and animal group (Control vs. C3 + BTX-A) as factors, showed significant main effects of TA l_MTU_ (*p* < 0.001) on passive forces, whereas no significant main effects of animal group (*p* = 0.33) and no significant interaction between factors (*p* = 0.98) were found.

Active muscle forces. The ANOVA results, with l_MTU_ values of the TA and animal group (Control vs. C3 + BTX-A) as factors, showed significant main effects of both factors on active forces (*p* < 0.001) but no significant interaction (*p* = 0.72). Compared to the Control group, muscle active forces were lower in the C3 + BTX-A group (on average by 43.5%). For l_range_, compared to that of the Control group (11.1 ± 1.58 mm), no significant differences were found for the C3 + BTX-A (9.84 ± 1.81 mm) group (*p* = 0.19).

For further details on the experimental data, see Supplementary Materials, Table S1. Note that for reference purposes, Figure 1 is supplemented with BTX-A group data extracted from previous work [17]. The BTX-A group showed 12.3% higher passive forces, muscle length-dependent reductions in active force at most muscle lengths studied (e.g., a 48.3% reduction at the muscle’s optimum length), and a 22.9% narrower l_range_ compared to the Control group.

### 2.2. Study 2: Effects of C3 Transferase Alone on Muscle Mechanics and Structure

**Figure 2:**
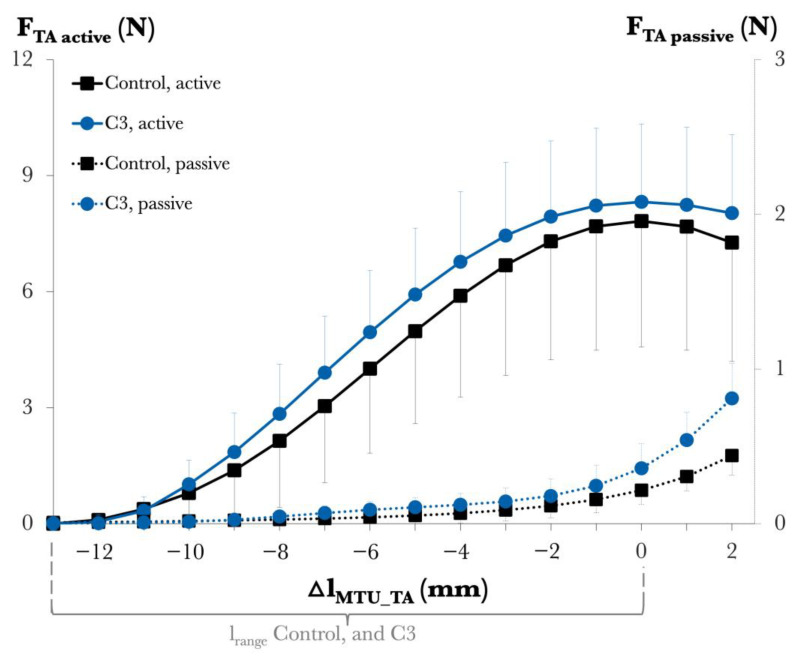
Forces of the tibialis anterior (TA) against its increasing muscle–tendon unit length (l_MTU_) in Study 2. Active and passive muscle forces are shown as mean values with standard deviations for the Control and C3 groups. l_MTU_ of the TA is expressed as a deviation from its optimum length (Δl_MTU_TA_ = 0 mm). For further details, see Supplementary Materials, Table S1.

Muscle mechanics. Figure 2 shows the TA muscle’s length–force characteristics. (1) Passive muscle forces. The ANOVA results, with l_MTU_ values of the TA and animal group (Control vs. C3) as factors, revealed a significant effect of TA l_MTU_ on passive forces (*p* < 0.001), no significant effect of the animal group (*p* = 0.09), and a significant interaction between factors (*p* < 0.01). The post hoc analysis showed significant effects of C3 transferase at the muscle optimum length and longer muscle lengths: for Δl_MTU_TA_ = 0, +1, and +2 mm, the uncorrected *p*-values for group comparisons were 0.01, 0.03, and 0.37, respectively, but after Bonferroni correction (0.23, 0.47, and 1.00), no comparisons remained significant. (2) Active muscle forces. ANOVA, with l_MTU_ of the TA and animal group (Control vs. C3) as factors, revealed a significant main effect of TA l_MTU_ (*p* < 0.001), whereas there was no significant main effect of the animal group (*p* = 0.28) and no significant interaction between factors (*p* = 0.34). Lastly, for l*_range_*, no significant differences were found between the Control group (11.1 ± 1.58 mm) and the C3 group (11.3 ± 1.28 mm) (*p* = 0.77).

**Figure 3:**
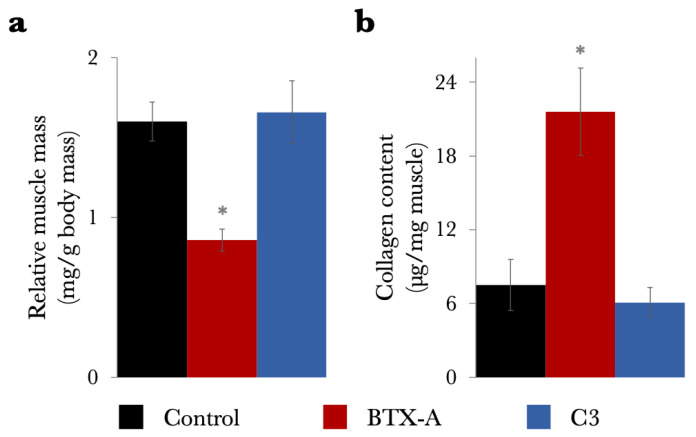
Muscle structure. (**a**) Muscle masses relative to the animals’ body masses and (**b**) collagen contents of the TA are shown as mean values with standard deviations for the Control, BTX-A, and C3 groups. Data concerning the BTX-A group were extracted from our earlier publication [17] to facilitate comparison and interpretation of the findings. The bars depict the comparison of the BTX-A and C3 groups to the Control group. Differences that are statistically significant compared to the Control group are indicated with *, specifically highlighting significant differences for BTX-A, not for C3. For further details, see Supplementary Materials, Table S1.

Muscle structure. (1) Muscle mass did not show a significant difference between the Control (0.65 ± 0.04 g) and C3 (0.75 ± 0.12 g) groups (*p* = 0.066). Further analysis, by normalizing muscle mass to the respective animal body mass (mg muscle/g body mass), yielded consistent results: no significant differences were found between the Control (1.60 ± 0.12 mg/g) and the C3 (1.66 ± 0.20 mg/g) groups (*p* = 0.298, Figure 3a). (2) Intramuscular collagen content did not show a significant difference between the Control (7.49 ± 2.08 µg collagen/mg) and the C3 (6.08 ± 1.22 µg collagen/mg) groups (*p* = 0.093, Figure 3b).

Note that for reference purposes, Figure 3 is supplemented with BTX-A group data extracted from previous work [17]. The BTX-A group showed a 46.4% lower relative muscle mass (0.86 ± 0.07 mg/g) and an almost three-fold (188.4%) increase in collagen content (21.61 ± 3.56 µg collagen/mg) compared to the Control group.

## 3. Discussion

The present findings demonstrate, for the first time, that supplementing BTX-A with C3 transferase mitigates the adverse effects of BTX-A on muscle mechanics in the rat [22]. Study 1 showed that the combined injection of BTX-A with C3 transferase into the rat TA results in (i) a reduction in active muscle forces, with no change in (ii) passive muscle forces and (iii) the muscle’s l_range_. This confirms our proposed hypotheses. The studied novel approach improved BTX-A effects on muscle mechanics by still leading to a substantial decrease in muscle tone while avoiding adverse outcomes, i.e., an increase in passive muscle forces and narrower muscle l_range_, which collectively represents elevated passive resistance coupled with limited muscle actuated mobility at the joint. Study 2, on the other hand, showed that C3 transferase administration alone does not alter muscle mechanics, induce muscle mass loss, or increase collagen content. Therefore, muscle atrophy and elevated muscle stiffness do not occur. Overall, these results provide a promising preclinical basis for the potential therapeutic value of integrating C3 transferase with BTX-A to enhance therapeutic outcomes by optimizing the main expected benefits of BTX-A while effectively minimizing its adverse effects on muscular mechanics and structure.

### 3.1. Injection Protocols in Experimental Models and Clinical Comparisons

Regarding C3 transferase injections, most of the literature focuses on cell culture studies, highlighting C3 transferase’s potency and utility in specifically blocking RhoA/B/C signaling. The only study that investigated fascia samples in vitro tested C3 transferase at 30 µg/mL (equivalent to 0.6 µg in 20 µL) and reported, acutely (3 h), a decrease in force in rat lumbar fascia [21]. In the present study, we applied a higher dose to investigate the effects at the whole muscle level and over a much longer duration. Regarding BTX-A injections, the safe dose range for children with CP is 1 to 25 U/kg bodyweight [23]. Clinical doses of BTX-A for lower extremity muscles typically range from 3 to 6 U/kg [24,25], depending on factors such as muscle volume, spasticity level, and the extent of muscle involvement in the patient’s joint movement pathology [26]. In contrast, the experimentally used BTX-A dose presently approximates only 0.32 U/kg. This is considerably lower than the clinical doses. However, this dose, at the volume injected, did yield an effective toxin distribution. Our results indicate substantial paralysis within the target muscle, which is in concert with previous animal studies [10,17]. Specifically, in rat TA muscle, a dose of 0.02 U (a fifth of the dose used presently) injected into the mid-belly can paralyze approximately one-fifth of the total cross-sectional area within just 24 h [27]. Additionally, the presently injected volume (approximately 64 µL/kg) is lower than the clinical volumes used (2.5–8 mL/kg) for lower limb muscles [24,25]. Overall, the following general limitation of animal studies, including the present one, is to be considered: the difficulty in directly correlating experimental doses and volumes with clinical practice. Yet, as in the present study, animal studies do serve the purpose of revealing unknown effects of tested chemicals that warrant further clinical investigation. The injected dose and volume are consistent with those used in our group’s BTX-A experiments [17]. This allowed us to conduct a direct comparison between different test conditions, including injections of BTX-A alone and with the addition of C3 transferase.

### 3.2. Addressing Muscle Atrophy and Growth Challenges

BTX-A is effective in reducing the development of permanent contractures in appropriate patient groups. Therefore, enhanced motor ability and a potential delay in the need for surgical interventions have been reported [28,29,30]. However, while the clinical outcomes of BTX-A therapy are well-documented, a notable gap in our understanding of the structural changes in the muscles exposed to BTX-A remains. Early animal studies highlight the need to identify the complex and sometimes contradictory effects of BTX-A on the muscular level [11,17,31,32,33]. Exposure to BTX-A appears to induce mechanical and structural changes in muscles, which may elevate similar effects typically associated with spasticity [4]. One primary concern is the onset of muscle atrophy and hindered muscle growth. Numerous studies have demonstrated a significant reduction in muscle mass due to BTX-A [11,17,33]. Reduced spasticity can largely be a secondary effect of such muscle atrophy [34], during which muscle contractile elements are partially replaced by fat and connective tissue [35]. Furthermore, research indicates that even after the paralysis effect of BTX-A subsides, the muscle’s morphological recovery is often incomplete [9,31]. Longitudinal studies in children with CP have reported persistent reductions in muscle volume and cross-sectional area, indicating compromised muscle growth even six months after injection [8,36,37]. In some cases, these changes can persist for up to one year [38]. For example, muscle growth in the medial gastrocnemius of children with spastic CP has been found to be approximately 60% lower than in age-matched typically developing peers [39]. Yet, it remains unclear whether this is a direct consequence of exposure to BTX-A or a reflection of intrinsically slower muscle growth rates in children with CP. Such impaired muscle development is recognized as a potential factor contributing to muscle weakness and motion limitations [40,41]. However, functional improvements are often observed following BTX-A treatment coupled with subsequent rehabilitation strategies such as physical therapy [42,43]. This plausibly benefits largely from the neurodenervation leading to a temporarily reduced muscle spasticity facilitating joint motion, but the changes to the muscular mechanics may not be under control. Importantly, the muscle structure analyses conducted presently reveal the following: C3 transferase alone neither causes any muscle mass loss nor leads to increased collagen content. This strongly suggests that elevated muscle stiffness is typically associated with BTX-A, and, unless mitigated, adverse mechanical effects are plausible.

### 3.3. Muscle Mechanical Changes Induced by Combined Injection

The combined injection of C3 transferase and BTX-A resulted in a significant decrease in active muscle forces (on average by 43.5%). Of note were the differences in the force reduction effect that were observed in BTX-A injection alone (maximally by 75.2% at short, and minimally by 48.3%, at long muscle lengths), which was muscle-length-dependent [10,17]. Such muscle length dependency of BTX-A-induced force reduction [44,45] is important to consider, as it may have significant clinical implications. This effect is ascribable to changes in the number of in-series sarcomeres [44] and/or changes in the heterogeneous distribution of the lengths of sarcomeres [15,16] along muscle fibers and within the muscle. For instance, sarcomere length heterogeneity at greater muscle lengths may result in some sarcomeres being positioned along both the ascending and descending limbs of their force–length curves within the same muscle fibers [46]. Consequently, at such lengths, a more complex mechanism may govern the effectiveness of BTX-A in reducing force. Importantly, when BTX-A injection is supplemented by C3 transferase, the decrease in active muscle force that we quantified is no longer dependent on muscle length. This suggests that C3 transferase may play a notable role in BTX-A-induced effects on muscular mechanics, mitigating length-dependent force reduction and, hence, potentially offering a more controllable therapeutic effect for managing spasticity in clinical settings. Understanding these dynamics can help optimize treatment strategies, ensuring better outcomes for patients by addressing the complex interplay between muscle length and force generation.

The mechanism of BTX-A’s effects on muscular mechanics, particularly regarding its reflection on muscle’s l_range_, has been studied using finite element modeling [15,16]. The characteristic determinant for this mechanism is the “longer sarcomere effect”. In short, inactivated muscle fibers modeled across half of the muscle represent BTX-A-induced partial muscle paralysis, and sarcomeres in those parts do not shorten on muscle activation. This also limits sarcomere shortening in the active muscle parts due to muscle fiber–ECM mechanical interactions, leading to an overall limited shortening of sarcomeres in the muscle exposed to BTX-A compared to their counterparts in a BTX-A-free muscle [15]. Due to such a longer sarcomere effect, the sarcomeres reach their maximal force production earlier, and this causes the muscle’s optimum length to shift to a shorter muscle length. Previous experimental studies have used this phenomenon to explain the narrowing of the l_range_ value observed after BTX-A injection [10,11,13,17,18]. Note that the narrower l_range_ effect was accompanied by a significantly increased total collagen content [11,13,17]. Therefore, the ECM structure did change, and this stiffening was apparent in the significantly elevated passive forces of the tested muscles exposed to BTX-A. This finding was used in the finite element modeling of the time course of treatment of BTX-A effects, and the results indicated that due to a stiffer ECM, the narrower l_range_ effect became even more pronounced in the long-term and partially persisted post-treatment [16]. Note also that the length-dependent muscle force reduction effect was also explained essentially via the muscle fiber–ECM mechanical interactions [16]. However, presently, the combined injection of C3 transferase and BTX-A removed those adverse effects that are typically associated with BTX-A and to which ECM stiffness is central. Nevertheless, while the unwanted BTX-A effects were eliminated, there was no visible improvement in the muscle’s contribution to the joint range of motion or a reduction in the passive resistance. As those effects are determined by the muscle ECM’s structural and mechanical properties [47,48] and intermuscular mechanical interactions [49,50,51,52], we suggest that future studies are needed to explore the effects of combining C3 transferase and BTX-A (i) at varying dosages and (ii) in different mechanical conditions to achieve clinically more relevant outcomes.

### 3.4. Limitations and Future Directions

While this study presents novel insights into the combined effects of BTX-A and C3 transferase, limitations must be acknowledged. First, as a preclinical study, a direct translation to clinical practice necessitates new studies, which need to take into account challenges stemming from species-based differences in muscle structure as well as neurodenervant injection parameters. Second, our study focuses on long-term effects post-injection, indicating that post-treatment effects of C3 transferase-supplemented denervant injection need to be tested in future studies. Third, although the present results do demonstrate clearly improved muscular mechanics, the underlying molecular mechanisms of those effects require further investigation. Note that BTX-A primarily targets SNARE proteins at neuromuscular junctions, and C3 transferase inhibits Rho GTPases, suggesting that direct biochemical interactions between BTX-A and C3 transferase are unlikely due to their distinct mechanisms of action. Yet, the possible effects of mixing these compounds require attention. For example, if this could partially limit BTX-A-induced muscle paralysis, it needs to be tested, although our findings did show that BTX-A in combination with C3 transferase yielded a major 43.5% reduction in muscle active force. Investigating, e.g., sarcomere architecture, ECM remodeling, and muscle fiber composition after exposure to BTX-A + C3 transferase will be essential to better understand effect mechanisms and, more importantly, to fine-tune dose and injection parameters to be suitable for clinical application processes. Finally, such understanding is also indicated to be complemented with in vivo studies for functional assessments of joint-level kinematics and kinetics.

## 4. Conclusions

The innovative application of BTX-A, combined with C3 transferase, studied for the first time presently, was shown to effectively retain the typical benefit of reduced muscle tone while also mitigating the adverse effects of BTX-A on muscle mechanics and preserving muscle structure. The results of the present preclinical study suggest that C3 transferase supplementation may offer a promising strategy for optimizing BTX-A therapy, e.g., in spasticity management. However, the need for more detailed investigations in animal models to better understand the effect mechanism and to refine the drug formulation is indicated. Of course, subsequently, the transition from animal testing to human studies conducted at the muscular as well as joint levels is essential.

## 5. Materials and Methods

### 5.1. Study 1: Assessment of Effects of Combined Administration of BTX-A and C3 Transferase on Muscle Mechanics

All methods were carried out in accordance with relevant guidelines and regulations. Animals were obtained from the Animal Care and Production Unit (LifeSci Vivarium, Boğaziçi University). All surgical and experimental procedures were approved by the Committee on the Ethics of Animal Experimentation at Boğaziçi University. Male Wistar rats were divided into two groups: Control (*n* = 7) and C3 + BTX-A (*n* = 7). The animal body masses for the Control and C3 + BTX-A groups, respectively, were 386.3 ± 36.5 g and 404.6 ± 17.9 g at the time of injection and 406.9 ± 16.8 g and 414.1 ± 27.9 g at the time of the experiment.

#### 5.1.1. Injection Protocol

After imposing mild sedation with an intraperitoneal ketamine injection (1 mg/kg), a circular region of approximately 15 mm radius from the center of the patella was shaved. The TA muscle was located by palpation with the ankle in maximal plantarflexion and the knee angle at 90°. After marking the center of the patella, a second marker was placed at a point 10 mm distally along the tibia. The injection location was 5 mm lateral (along the line segment drawn between the two markers) to the second marker and over the TA muscle. All injections were made exclusively into this muscle, to a depth of 3 mm. Note that, at the site of injection, the depth of the TA was approximately 5–5.5 mm, whereas the thickness of the skin was approximately 0.7–1 mm. Therefore, injections were made 1 month before testing into the superficial half of the TA muscle. All injected volumes equaled 20 µL (see below). To prevent infections and ensure reliable results, aseptic conditions were maintained during the injection procedure. After injection, the animals were housed individually in standard cages with free access to normal activity. The cages were maintained in a thermally regulated animal care room with a 12 h dark–light cycle for a month until the experiment day.

For the C3 + BTX-A group, a 100 U vial of vacuum-dried, botulinum type A neurotoxin complex (BOTOX, Allergan Pharmaceuticals, Westport, Ireland) and a 25 µg lyophilized powder of C3 transferase (C3 Transferase Protein–Bacterial recombinant, Cytoskeleton Inc., Denver, CO, USA) were separately reconstituted with normal (0.9%) saline solutions. Subsequently, a dose of 0.1 U/10 µL BTX-A was mixed with a dose of 2.5 µg/10 µL C3, and the animals received a one-time intramuscular injection of this mixture with a total dose of 0.1 U BTX-A and 2.5 µg C3 in 20 µL. The Control group received the same volume of normal saline solution exclusively.

#### 5.1.2. Surgical Procedure

The animals were anesthetized using an intraperitoneally injected urethane solution (1.2 mL of 12.5% urethane solution per 100 g of body mass). Additional doses were given if necessary (maximally 0.5 mL). Immediately following the experiments, the animals were euthanized by using an overdose of urethane solution.

During surgery and data collection, the animals were kept on a heated pad (Homoeothermic Blanket Control Unit; Harvard Apparatus, Holliston, MA, USA) to prevent hypothermia. A feedback system utilizing an integrated rectal thermometer allowed for the control of the body temperature at 37 °C by adjusting the temperature of the heated pad.

The skin and the biceps femoris muscle of the left hindlimb were removed, and the anterior crural compartment, including the extensor digitorum longus (EDL), TA, and extensor hallucis longus (EHL) muscles, was exposed. Only a limited distal fasciotomy was performed to remove the retinaculum (i.e., the transverse crural ligament and the crural cruciate ligament). The connective tissues of the muscle bellies within the anterior crural compartment were left intact.

The combination of knee joint and ankle angles (120° and 100°, respectively) was selected as the reference position corresponding to an in vivo position at the stance phase of the rat during gait [53]. In the reference position, the four distal tendons of the EDL muscle were tied together using silk thread. Matching markers were placed on the distal tendons of the EDL, TA, and EHL muscles, as well as in a fixed location on the lower leg. Subsequently, the distal EDL tendon complex, as well as the TA and the EHL tendons, were cut as distally as possible.

The femoral compartment was opened for two purposes: (1) to reach the proximal tendon of the EDL; after reaching it, the tendon was cut from the femur with a small piece of the lateral femoral condyle still attached, and (2) to expose the sciatic nerve. After this was completed, the sciatic nerve was dissected free of other tissues, and all nerve branches to the muscles of the femoral compartment were cut. Subsequently, the sciatic nerve was cut as proximally as possible.

To provide connection to force transducers, Kevlar threads were sutured to (1) the proximal tendon of the EDL muscle, (2) the tied distal tendons of the EDL muscle, (3) the distal tendon of the TA muscle, and (4) the distal tendon of the EHL muscle.

#### 5.1.3. Experimental Setup and Procedure

**Figure 4:**
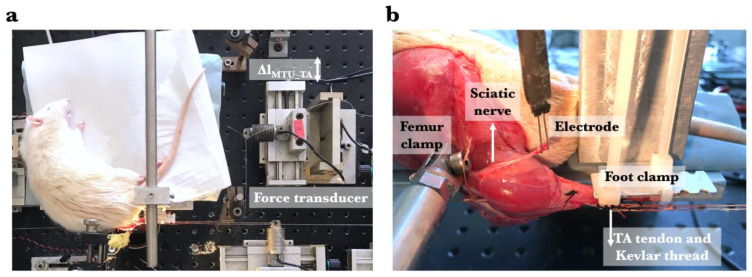
The experimental setup. (**a**) The distal tendon of the TA muscle was individually connected to a force transducer via a Kevlar thread. Throughout the experiment, the lengths of other compartmental muscles remained constant, while, exclusively, the TA muscle (MTU: muscle–tendon unit) was progressively lengthened (Δl_MTU_TA_) to increasing lengths, at which isometric contractions were performed. Lengthening (indicated by the double-headed arrow) started from muscle active slack length at 1 mm increments by adjusting the position of the TA force transducer. (**b**) Joint angles were standardized at 120° for the knee and 100° for the ankle, serving as the experimental reference position. Metal clamps were utilized to secure the femur and the foot in place. The distal end of the sciatic nerve was placed on a bipolar silver electrode.

Each animal was mounted in the experimental setup (Figure 4a). The femur and foot were fixed with metal clamps such that the ankle was in maximal plantar flexion (180°) to allow for the free passage of the Kevlar threads to the distal force transducers. The knee angle was set at 120°. Each Kevlar thread was connected to a separate force transducer (BLH Electronics, Inc., Canton, MA, USA). Care was taken to ensure the alignments of the Kevlar threads were in the muscle line of pull. The distal end of the sciatic nerve was placed on a bipolar silver electrode (Figure 4b). The room temperature was kept at 26 °C. For the duration of the experiment, muscles and tendons were irrigated regularly with isotonic saline to prevent dehydration.

The distal and proximal tendons of the EDL and the distal tendon of the EHL muscles were kept in their reference positions at all times during the experiment. Muscle isometric forces were measured simultaneously at various muscle–tendon unit lengths (l_MTU_) of the TA. Starting from its active slack length, the TA length was increased by moving its force transducer (in increments of 1 mm) until reaching 2 mm over its optimum length. TA muscle–tendon unit lengths are expressed as deviation from its active slack length (Δl_MTU_TA_).

AcqKnowledge software (version 3.9; BIOPAC Systems, Inc., Goleta, CA, USA) was used for data acquisition. The compartmental muscles were activated maximally by supramaximal stimulation of the sciatic nerve (STMISOC; BIOPAC Systems, Inc., Goleta, CA, USA) using a constant current of 2 mA (square pulse width 0.1 ms). After setting the TA muscle to a target length, two twitches were evoked, and 300 ms after the second twitch, the muscles were tetanized (pulse train 400 ms, frequency 100 Hz). At 200 ms after the tetanic contraction, another twitch was evoked. After each application of this stimulation protocol, the muscles were allowed to recover for 2 min. Recovery was allowed to occur near the TA’s active slack length.

### 5.2. Study 2: Assessment of the Effects of C3 Transferase Alone on Muscle Mechanics and Structure

In a separate set of male Wistar rats, changes in muscle mechanics and structure (i.e., muscle mass and intramuscular connective tissue content) were studied: Control (*n* = 6) and C3 (*n* = 6). The animal body masses for the Control and C3 groups, respectively, were 389.3 ± 37.3 g and 419.8 ± 49.5 g at the time of injection and 404.3 ± 31.0 g and 447.7 ± 28.8 g at the time of the experiment.

The incision protocol was kept the same as in Study 1. The Control group received 20 µL of normal saline solution exclusively, whereas, for the C3 group, a 25 µg lyophilized powder of C3 transferase was reconstituted with a normal (0.9%) saline solution, and the animals received a one-time intramuscular injection with a total dose of 2.5 µg C3 in 20 µL.

The injection as well as surgical and experimental procedures were identical to those applied in Study 1. In addition to muscle mechanics testing, muscle structure was also evaluated by quantifying the intramuscular collagen content using a colorimetric hydroxyproline assay [54]. Muscle biopsies were removed rapidly after euthanizing each animal and weighed. The purity of the muscle samples was ensured by careful removal of all tendinous structures from the sample. The muscle biopsies were flash-frozen in liquid nitrogen and stored at −80 °C before running the assay within 4 weeks after removal. For analysis, each muscle sample underwent hydrolyzation at 130 °C for 12 h in 5 N HCl. The samples of the hydrolyzate were oxidized at room temperature with a chloramine-T solution for 25 min incubation. Subsequently, the impurities were extracted and discarded by toluene treatment. To convert the oxidation product to pyrrole, the remaining aqueous layer containing the hydroxyproline products was heated for 30 min in boiling water. The final pyrrole reaction product was then removed in a second toluene extraction, and the final solution was mixed with Ehrlich’s reagent for 30 min. Sample absorbances were read at 560 nm in triplicate using a UV–visible spectrophotometer (UV-1280; SHIMADZU, Kyoto, Japan).

### 5.3. Data Processing

Isometric passive muscle forces. The forces measured 100 ms after the second twitch, corresponding to the muscles’ inactive state (*F_p_*), were studied to characterize the mechanical resistance capacity of muscular tissues at the l_MTU_ tested.

Isometric active muscle forces. The mean forces for a 200 ms interval during the tetanic plateau, 150 ms after evoking tetanic stimulation, were determined as the muscle’s total force. Active muscle forces (*F_a_*) were calculated by subtracting the measured passive force from the measured total force per l_MTU_.

Data for *F_p_* and *F_a_* were fitted with a polynomial function using a least squares criterion:

(1) $$y=a_{0}+a_{1}x+a_{2}x^{2}+\cdots +a_{n}x^{n}$$

where y represents isometric muscle forces (i.e., *F_p_* or *F_a_*) and x represents the l_MTU_. a_0_, a_1_…a_n_ are coefficients determined in the fitting process.

The lowest order of the polynomials that still added a significant improvement to the description of changes in l_MTU_ and muscle force data were selected using one-way analysis of variance (ANOVA) [55]. These polynomials were used for averaging data and calculation of standard deviations. TA muscle forces at different l_MTU_ were obtained by using these functions. For each l_MTU_, TA forces were averaged, and standard deviations were calculated to determine the muscle force (mean ± SD) of the Control and experimental groups.

The major determinants of muscle length–force characteristics are muscle optimal force (the maximum isometric force exerted by an active muscle), the corresponding muscle optimum length, and muscle active slack length (the shortest length at which the muscle can still exert nonzero force). Muscle optimal force is often taken as an indication of a muscle’s capacity for force production, and the range from active slack length to the length at which optimal force is exerted is taken as an indicator of movement capability with active force exertion within the potential range of motion of a certain joint. To account for these issues, the polynomials obtained were also used to determine (i) the optimal TA force (i.e., for each TA muscle, the maximum value of the fitted polynomial for active muscle force) and the corresponding muscle length and (ii) TA active slack length. The TA length range of active force exertion (l_range_) was determined as the range between the active slack length and the length at which optimal force was measured.

Hydroxyproline analysis was used to measure changes in intramuscular collagen content caused by C3 transferase per se. Using the measured absorbance values of the muscle samples, the hydroxyproline contents of individual muscles were determined based on a reference (i.e., standard regression curve identifying the paired information of previously known hydroxyproline amounts and their measured absorbance values) as mg hydroxyproline expressed per mg of muscle tissue wet weight. Hydroxyproline content was converted into intramuscular collagen content using a constant (7.46), which characterizes the number of hydroxyproline residues in one molecule of collagen [56].

### 5.4. Statistical Analyses

Statistical analyses were conducted using MATLAB R2023a (The MathWorks, Inc., Natick, MA, USA). A two-way repeated measures ANOVA (Study 1) or a two-way mixed ANOVA with Satterthwaite’s method for degrees of freedom and Type III sum of squares (Study 2) was performed to detect the effects of the main factors (i.e., TA l_MTU_ and animal group) on TA forces. If significant main effects were found, Bonferroni post hoc tests were performed to further locate significant within-factor differences.

Group forces were aligned for their optimum lengths. Changes in active force were determined by computing the difference between the mean force observed within the experimental group (C3 + BTX-A or C3) across each l_MTU_ of the TA, relative to that of the Control group. The differences were then expressed as a percentage of the mean force exhibited by the Control group.

The Shapiro–Wilk normality test was used to check if l_range_, (relative) muscle mass, and collagen content data were normally distributed. Accordingly, the TA’s l_range_, (relative) muscle mass, and collagen amount calculated for each muscle in the experimental groups were compared to those of the Control group using unpaired-t or Mann–Whitney U test, where appropriate. All differences were considered significant at *p* < 0.05.

Data concerning the BTX-A group were extracted from our earlier publication [17] to facilitate comparison and interpretation of the findings.

## 6. Patents

We acknowledge that the new botulinum toxin formula described in this manuscript is protected by a patent (publication number: WO/2023/172230, publication date: 14 September 2023, available online: https://patentscope.wipo.int/search/en/detail.jsf?docId=WO2023172230&_cid=P22-LO8EN5-90918-1 (accessed on 10 April 2025)) filed by C.Y. and C.K.

## Data Availability

The original contributions presented in this study are included in this article and Supplementary Materials. Further inquiries can be directed to the corresponding author.

## Supplementary Materials

The following supporting information can be downloaded at: https://www.mdpi.com/article/10.3390/toxins17050234/s1, Table S1: experimental data.

## Abbreviations

The following abbreviations are used in this manuscript:

ANOVA	analysis of variance
BTX-A	botulinum toxin type A
CP	cerebral palsy
ECM	extracellular matrix
EDL	extensor digitorum longus
EHL	extensor hallucis longus
TA	tibialis anterior
